# ApiRegenin, an Animal-Derived Platelet-Rich Plasma Extract, Accelerates Wound Healing of Chronic Diabetic Ulcer in Mice

**DOI:** 10.3390/pharmaceutics18070856

**Published:** 2026-07-14

**Authors:** Zheng-Qi Wang, Minnie Wing-Yi Mak, Xiong Gao, Yu-Tong Ye, Christina Lok-Pan Yik, Tina Ting-Xia Dong, Karl Wah-Keung Tsim

**Affiliations:** 1 Center for Chinese Medicine, Division of Life Science, The Hong Kong University of Science and Technology, Clear Water Bay, Kowloon, Hong Kong 999077, China; zwangie@connect.ust.hk (Z.-Q.W.); wymakac@connect.ust.hk (M.W.-Y.M.); gaoxiong@ust.hk (X.G.); yyebl@connect.ust.hk (Y.-T.Y.); lpyik@connect.ust.hk (C.L.-P.Y.); botina@ust.hk (T.T.-X.D.); 2 Shenzhen Key Laboratory of Edible and Medicinal Bioresources, HKUST Shenzhen Research Institute, Shenzhen 518057, China

**Keywords:** platelet-rich plasma, chronic diabetic wound, macrophage polarization, angiogenesis

## Abstract

**Background:** Platelet-rich plasma (PRP) plays a crucial role in chronic wound healing by releasing growth factors that regulate inflammation, promote angiogenesis, and stimulate tissue regeneration. **Methods and Results:** Here, an animal source of PRP, named ApiRegenin and derived from cultivated deer blood, was established. Specific protein and non-protein biomarkers—including nicotinamide, palmitic acid, IGF, and fibronectin—were validated to ensure batch-to-batch quality control. The pharmacological properties of ApiRegenin in cultured cells transfected with DNA encoding HRE and NF-κB reporter constructs were validated, serving as a functional control. In a skin-defective model of *db/db* diabetic mice, accelerated wound healing was observed following ApiRegenin treatment. Histological analysis revealed enhancements of re-epithelialization, granulation tissue formation, and collagen deposition. In parallel, the immunofluorescence staining of CD31, α-SMA, and VEGF was upregulated, indicating the promotion of angiogenesis. Furthermore, ApiRegenin treatment shifted the local immune microenvironment toward an M2-like macrophage phenotype, characterized by the downregulation of iNOS and the contrastive upregulation of Arg-1. At the molecular level, transcriptomic enrichment analysis suggested the prominent involvement of the HIF-1, PI3K-Akt, and TNF signaling pathways. **Conclusions:** These findings demonstrate that ApiRegenin effectively accelerates diabetic wound healing by promoting angiogenesis and modulating macrophage polarization.

## 1. Introduction

Chronic diabetic wounds are a severe complication associated with numerous pathophysiological conditions, characterized by persistent inflammation, impaired tissue repair, and infection [1]. Approximately 25% of diabetic patients develop chronic skin ulcers, of which diabetic foot ulcers account for 15% to 25% [2,3]. The yearly amputation rate for diabetic foot ulcers can be as high as 22%, while the annual mortality rate is around 11% [2]. This unmet medical need is attributed not only to the direct toxicity of hyperglycemia but also to the interplay of multiple factors, including neuropathy, vascular complications, oxidative stress, and immune dysregulation [4]. The management of chronic diabetic wounds aims to promote healing, prevent infection, and eliminate inflammation. Currently, the main treatments include debridement and infection control, hyperbaric oxygen therapy, and revascularization as basic interventions [5]; however, these basic interventions often face issues, such as limited efficacy, significant risks, or high treatment costs, making it difficult to achieve fundamental healing of the wound [6,7].

Human platelet-rich plasma (PRP) refers to the plasma fraction concentrated from blood platelets after centrifugation [8], which is known to contain various growth factors [9,10]. The growth factors and fibrinogen in PRP effectively promote tissue regeneration and wound healing [11,12]. However, the clinical use of autologous human PRP is hindered by several key constraints, including the limited blood supply available from individual donors, the substantial expenses associated with its preparation, and the requirements of rigorous regulatory standards.

Animal-derived PRP has gained attention as an alternative source, offering immediate availability and scalability for experimental and therapeutic applications [13]. In animals, PRP compositions and their activities vary considerably across species, influencing both their efficacy and safety profile [14]. Indeed, PRP from multiple animal sources has been researched for antioxidant activity, wound-healing capacity, and anti-inflammatory effects [15]. Furthermore, the effectiveness of PRP is highly dependent on the preparation methodologies and interspecies variations in hematological indices. Preparative parameters, such as centrifugal force, duration, and processing techniques, critically influence the platelet recovery, volume, and functional integrity [16]. To address these challenges, the extraction process of PRP was optimized by systematically evaluating various parameters, including the centrifugal force, freeze–thaw cycles, sonication, and calcium chelators [10]. The PRP derived from deer was optimized by a unique extraction procedure, as described before [17]. Following a rigorous series of functional validations—including anti-inflammatory and wound-healing assays—alongside safety evaluations encompassing its immunostimulatory potential and the Ames bacterial reverse mutation test, the resulting formulation was designated as “ApiRegenin”. To develop ApiRegenin as a drug for clinical application, we established quality control parameters for the isolation of ApiRegenin, including biomarker qualification and functional profiling. Moreover, the therapeutic efficacy of ApiRegenin was assessed in a wound model from diabetic mice. These findings provide a foundation for the clinical use of ApiRegenin as a promising therapeutic agent for chronic wound management.

## 2. Materials and Methods

### 2.1. Materials

The Amplex Red Free Fatty Acid Assay Kit was purchased from Beyotime (Shanghai, China; Cat. No. S0215S). Primary antibodies were purchased from Abcam (Cambridge, UK), including anti-VEGF (Cat. No. ab69479), anti-CD31 (Cat. No. ab28364), anti-Arg 1 (Cat. No. ab233548), anti-iNOS (Cat. No. ab178945), and anti-α-SMA (Cat. No. ab7817). RNAzol^®^ RT reagent (Cat. No. R4533), dimethyl sulfoxide (DMSO) (Cat. No. D2650), and other routine chemicals were purchased from Sigma-Aldrich (St. Louis, MO, USA). Insulin-like growth factor (IGF) (Cat. No. KS19942) and fibronectin (Cat. No. KS20419) ELISA kits were purchased from Yanshunbio (Guangzhou, China).

### 2.2. Preparation of ApiRegenin

Blood samples were obtained from cultivated red deer (*Cervus elaphus*) originating from Gansu, China. Harvested deer blood was centrifuged at 200× *g* for 10 min at 4 °C to separate the PRP, followed by a second centrifugation at 1200× *g* for 15 min to concentrate the platelets, as described previously [10,17]. The concentrated platelets underwent three consecutive freeze–thaw cycles consisting of freezing at −80 °C for 2 h followed by rapid thawing in a 37 °C water bath for 10 min. The formulation was exposed to short-wave ultraviolet light for 15 min in a laminar flow hood to inactivate potential viral contaminants before being freeze-dried.

### 2.3. Enzyme-Linked Immunosorbent Assay (ELISA)

The levels of IGF and fibronectin in ApiRegenin were quantified based on a previously optimized protocol [18]. In brief, ApiRegenin specimens from various batches were added to a coated plate and incubated for 30 min. Capture antibodies and detection antibodies were added successively, followed by a further 2 h incubation period. After multiple washing steps to eliminate unbound substances, a chromogenic substrate solution was introduced into each well, and the reaction was halted after 10 min of incubation in the dark. The absorbance at 450 nm was detected.

### 2.4. Palmitic Acid Analysis

The quantification of palmitic acid in ApiRegenin was conducted using the amplex red free fatty acid assay kit. To prepare a 100 µM standard solution, 20 µL of the 1 mM palmitic acid standard solution was added to 180 µL of buffer. Aliquots of 0, 5, 10, 20, 30, 40, and 50 µL of this solution were added to a 96-well plate and adjusted to a final volume of 50 µL with buffer, for the establishment of a standard curve. For sample wells, 1–50 µL of diluted test sample was added, and each well was brought to 50 µL with buffer. A blank control well containing only buffer was prepared. Subsequently, 2 µL of enzyme mixture B was added to each test well and 2 µL of detection buffer to the control well; after gentle mixing, the plate was incubated at 37 °C in the dark for 30 min, followed by the addition of 50 µL working solution per well. Following another round of mixing and 30 min dark incubation at 37 °C, the absorbance at 570 nm was measured with a microplate reader, and the palmitic acid concentration was quantified based on the standard curve.

### 2.5. LC-MS/MS Analysis of Nicotinamide

Analysis of nicotinamide was performed using an Agilent 6410 triple-quadrupole mass spectrometer coupled with a Luna^®^ HILIC column (100 mm × 2 mm, 200 Å, 3 µm) [19], with an injection volume of 5 µL; mobile phase A was water containing 0.1% formic acid and mobile phase B was acetonitrile with 0.1% formic acid, and gradient elution was conducted over 14 min following a programmed gradient (0–1 min: 55% B isocratic; 1–1.3 min: linear decrease to 35% B; 1.3–2.5 min: 35% B isocratic; 2.5–2.51 min: linear increase to 100% B; 2.51–4 min: 100% B isocratic; 4–4.01 min: linear decrease to 55% B; 4.01–6 min: 55% B isocratic; 6–14 min: column re-equilibration), with niacinamide retention time at approximately 2.2 min; the mass spectrometer was operated in positive electrospray ionization (ESI^+^) mode with a capillary voltage of 3.5 kV, cone voltage of 10 V, ion source temperature of 100 °C, desolation temperature of 325 °C, and ultra-high-purity nitrogen as the nebulizing gas (10 L/min, 40 psi), full-scan mass spectra were recorded at *m*/*z* 50–1000, and multiple reaction monitoring (MRM) was adopted with niacinamide precursor ions [M+H]^+^ at *m*/*z* 123.1, product ions at *m*/*z* 80.0 (quantitative) and *m*/*z* 96.0 (qualitative) after 15 eV collision-induced dissociation and niacinamide d4 as internal standard (transition *m*/*z* 127.1 → 84.0); for sample preparation, ApiRegenin samples were diluted and filtered through a 0.22 µm membrane prior to LC-MS/MS analysis.

### 2.6. Cell Culture and Viability Test

The human keratinocyte HaCaT cell line (American Type Culture Collection, ATCC, Manassas, VA, USA) was cultured in DMEM medium supplemented with 10% fetal bovine serum (FBS) and 1% penicillin/streptomycin (100 U/mL and 100 µg/mL) in a humidified incubator at 37 °C with 5% CO_2_. Cell viability was evaluated using the MTT [3-(4,5-dimethyl-2-thiazolyl)-2,5-diphenyl-2H-tetrazolium bromide] assay, as previously reported [20]. Cells were seeded into 96-well plates and incubated for 24 h prior to treatment with ApiRegenin for another 24 h. Subsequently, MTT solution (5 mg/mL) was added to each well, and the cells were incubated at 37 °C for 4 h. After removal of the culture medium, the formazan crystals formed in viable cells were dissolved in DMSO, followed by shaking for 15 min. The absorbance was measured at 570 nm using a microplate reader.

### 2.7. DNA Transfection and Luciferase Assay

Cultured cells were transfected with pNF-κB-Luc and pHRE-Luc reporter plasmids based on a previously verified method [21]. HaCaT keratinocytes were pretreated with ApiRegenin prior to stimulation with 20 ng/mL TNF-α for 4 h. Dexamethasone (Dex, 10 nM) and tert-butylhydroquinone (tBHQ, 20 µM) were utilized as positive controls for pNF-κB-Luc and pHRE-Luc vectors, respectively. After transfection and stimulation, cells were lysed and centrifuged. Aliquots of 20 µL supernatant were transferred to a 96-well assay plate, and luminescence intensity was detected. Bioluminescent signals were normalized to the total protein concentration of each cell lysate to reduce errors, as caused by protein content differences.

### 2.8. Diabetic Wound-Healing Model

Diabetic *db/db* male mice and wild-type C57BL/6 mice (all at 6–8 weeks old) were utilized for the full-thickness skin wound model. All experimental procedures were approved by the DSC institutional animal care and use committee under Protocol #24-018a1. Mice were anesthetized with pentobarbital (30 mg/kg), and their dorsal fur was shaved and disinfected. Two full-thickness wounds measuring 8 mm in diameter were created on the dorsum using a biopsy punch. The mice were divided into five treatment groups (*n* = 12): PBS (control); ApiRegenin at 40 mg/mL (Api-low); ApiRegenin at 200 mg/mL (Api-high); ON101 at 1250 mg/mL (ON101); and wild-type mice (wild type). Treatments were administered daily with 50 μL of the respective solutions. Following treatment, mice were housed individually in single cages, and the wounds were left uncovered. The dose of ON101 was based on the published paper [22]. The wound areas were photographed on days 0, 3, 5, 7, 10, 12, and 14 and analyzed using ImageJ software (version 1.53; National Institutes of Health, Bethesda, MD, USA). Wound closure was calculated with the formula:

$$Wound\;Closure\;Area=\frac{A_{0}-A_{t}}{A_{0}}\times 100\%$$

where *A*_0_ and *A_t_* are the initial wound area and the residual wound area, respectively, at each time point.

### 2.9. Histological Analysis

On days 7 and 14 post-operation, half of the experimental animals were euthanized to collect tissue blocks containing the entire wound area and at least 2 mm of adjacent normal skin; all specimens were fixed in 4% paraformaldehyde (pH 7.4) for 24 h, dehydrated through a graded ethanol series (70%, 80%, 95%, 100%) for 45 min per concentration, cleared twice with xylene (20 min each), embedded in paraffin, and sectioned into 4 μm serial slices, which were flattened in a 40 °C water bath, mounted on adhesive slides, and heated at 60 °C for 2 h to ensure firm adhesion. For histological staining, paraffin sections were dewaxed, rehydrated, and subjected to hematoxylin and eosin (H&E) staining (hematoxylin staining for 8 min, eosin counterstaining for 3 min) and Masson’s trichrome staining (Bouin’s solution fixation, Weigert’s iron hematoxylin nuclear staining, acid fuchsin and aniline blue differential staining); the stained sections were observed under a light optical microscope. Image Pro Plus 8.0 software was used to quantitatively analyze epidermal regeneration, granulation tissue thickness, epithelialized distance, collagen volume fraction and fiber arrangement to evaluate wound-healing and tissue remodeling quality.

### 2.10. Immunohistochemical (IHC) Staining

On days 7 and 14 post-surgery, wound tissues were harvested from diabetic mice, fixed in 4% paraformaldehyde for 24 h, dehydrated via a graded ethanol series, and embedded in paraffin to prepare 4 μm thick continuous sections; after dewaxing and rehydration, sections were treated with 3% H_2_O_2_ for 10 min to block endogenous peroxidase activity, subjected to antigen retrieval via high-pressure heating in citrate buffer (pH 6.0) for 2 min, blocked with 5% bovine serum albumin (BSA) at room temperature for 30 min, then incubated overnight at 4 °C with rabbit primary antibodies against α-SMA (1:200, Abcam), CD31 (1:100, Abcam), VEGF (1:150, Abcam), Arg 1 (1:200, Abcam) and iNOS (1:100, Abcam), followed by incubation with horseradish peroxidase (HRP)-conjugated goat anti-rabbit IgG polymer at 37 °C for 30 min, color development with 3,3′-diaminobenzidine (DAB) for 5–10 min, hematoxylin nuclear counterstaining, gradient dehydration, xylene clearing and neutral resin mounting for microscopic observation. For quantitative analysis, three non-continuous sections were selected per mouse, four to five high-power fields were randomly captured per section, and Image Pro Plus 8.0 software was used to quantify the average optical density of α-SMA, Arg 1, iNOS and VEGF positive expression, as well as count CD31-positive microvessel density.

### 2.11. RNA Sequencing and Bioinformatic Analysis

Total RNA was isolated from skin tissues with TRIzol^®^ Reagent following the manufacturer’s protocol, RNA purity was verified by the A260/A280 absorbance ratio using a Nanodrop ND 2000 spectrophotometer (Thermo Scientific, Wilmington, DE, USA) and RNA integrity number (RIN) was assessed with an Agilent Bioanalyzer 4150 system (Agilent Technologies, Santa Clara, CA, USA), with only qualified RNA samples (RNA Integrity Number, RIN ≥ 7.0) used for library construction; for each group, three independent biological replicates (*n* = 3) were processed. Paired-end sequencing libraries were prepared with the VAHTS Universal V10 RNA-seq Library Prep Kit for Illumina (Vazyme, Nanjing, China) per the manufacturer’s instructions, involving oligo (dT) magnetic bead enrichment of mRNA from 1 μg total RNA, mRNA fragmentation with divalent cations at high temperatures in ABclonal first-strand synthesis buffer, synthesis of first-strand cDNA with random hexamer primers and RNase H minus reverse transcriptase, generation of second-strand cDNA with DNA polymerase I, RNase H, reaction buffer and dNTPs, ligation of double-stranded cDNA fragments with sequencing adapters, PCR amplification for library enrichment, purification of amplified PCR products and validation of library quality via the Agilent Bioanalyzer 4150 system. High-throughput sequencing was executed on an Illumina NovaSeq/MGISEQ T7 platform, yielding an average read depth of ≥45 M raw reads per sample.

Following sequencing, raw reads were quality-filtered using FastQC. The clean reads, which achieved a high mapping rate of 92.3–94.1%, were aligned to the mouse reference genome (GRCm39) using HISAT2. Gene expression reading counts were quantified using FeatureCounts and expression levels were normalized via the Fragments Per Kilobase million (FPKM) method. To control the false-discovery rate from multiple testing, *p*-values were adjusted using the Benjamini–Hochberg false-discovery rate (*FDR*) correction. The criteria for identifying differentially expressed genes (DEGs) were strictly defined as |log_2_ (Fold Change)| > 1 and adjusted *p*-value (*FDR*) < 0.05. Gene Ontology (GO) annotation analysis and Kyoto Encyclopedia of Genes and Genomes (KEGG) pathway enrichment analysis (https://www.genome.jp/kegg/ (accessed on 26 March 2025)) were subsequently conducted to evaluate the functional enrichment of DEGs and suggest potential intergroup pathways.

### 2.12. Statistical Analysis

All data were presented as the mean ± standard error of the mean (SEM). Statistical analyses were performed using two-way repeated-measures analysis of variance (ANOVA), one-way ANOVA, or mixed-effects analysis with GraphPad Prism 8.0.1 software (GraphPad Software Inc., San Diego, CA, USA). To conduct multiple pairwise comparisons, Bonferroni’s post hoc test was utilized following the ANOVA or mixed-effects procedures. In all comparative tests, significant differences relative to the basal or control group were defined as follows: * *p* < 0.05; ** *p* < 0.01; *** *p* < 0.001.

## 3. Results

### 3.1. Quality Assessment of ApiRegenin

**Figure 1:**
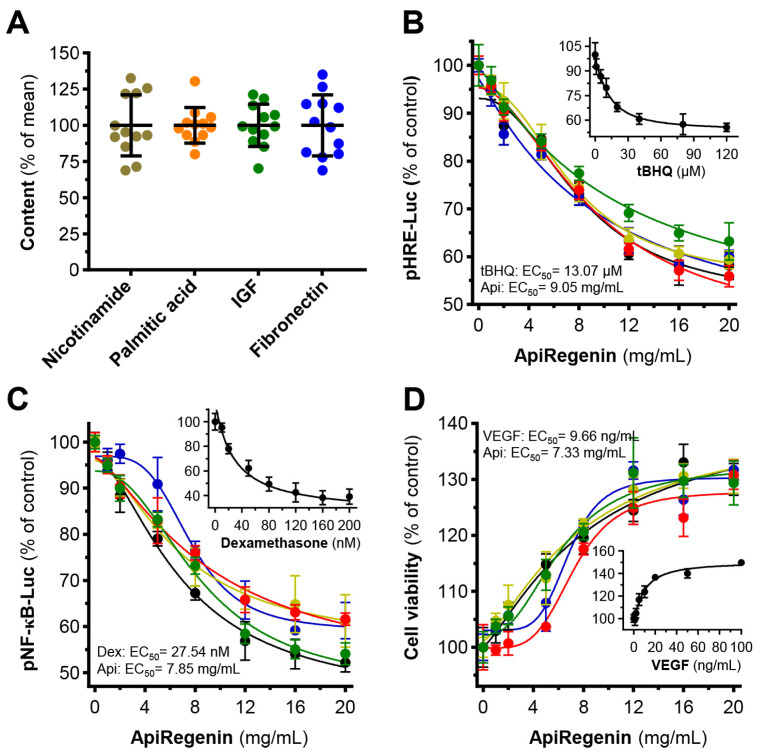
Quality assurance of deer PRP-ApiRegenin. (**A**) Twelve batches of ApiRegenin were analyzed for their amounts of nicotinamide (by LC-MS), palmitic acid (by amplex red free fatty acid assay kit), IGF (by ELISA), and fibronectin (by ELISA). The 100% on the y-axis corresponds to the mean content per one gram of dried ApiRegenin for each component, i.e., nicotinamide 0.16 ± 0.03 mg, palmitic acid 14.60 ± 1.80 mg, IGF 2.95 ± 0.43 µg, and fibronectin 1.12 ± 0.24 mg (*n* = 12). (**B**) The pHRE-Luc plasmid was transfected into HaCaT cells for 4 h, followed by pretreatment with tBHQ or ApiRegenin for an additional 4 h, and the cells were then exposed to TNF-α (20 ng/mL) for 20 h. Luciferase activity in each well was normalized to total protein content, and the results were expressed as a percentage of the normalized basal activity in untreated cells, which was set at 100%. Different colors of lines correspond to different batches of prepared ApiRegenin (*n* = 5). (**C**) The pNF-κB-Luc plasmid was transfected into keratinocyte cells for 4 h, followed by pretreatment with dexamethasone or ApiRegenin for 4 h, before exposure to TNF-α (20 ng/mL) for 20 h. Determination of luciferase activity was done as in (**B**). (**D**) VEGF, or ApiRegenin, was applied onto cultured keratinocytes for 24 h, and an MTT assay was determined. The values are expressed as a percentage of the normalized basal activity (untreated cells), set at 100%. The values are expressed in mean ± SEM, *n* = 3. The EC_50_ values are shown in the figures.

ApiRegenin derived from different batches of deer platelet-rich plasma was subjected to strict quality control assays. Roughly 1 g of ApiRegenin was extracted from 100 mL of deer blood, and this extraction yield was consistent with that of human blood as reported [10]. The metabolomics and proteomics were performed on isolated ApiRegenin indicating numerous possible active ingredients that could serve as biomarker quantification for the product [17]. As an active form of vitamin B3, nicotinamide acts as a critical precursor for the indispensable coenzymes NAD+ and NADP+, thus exerting a pivotal regulatory role in cellular metabolic processes and cutaneous wound healing. Nicotinamide facilitates tissue repair by providing energy, reducing inflammation, promoting cell proliferation, and restoring barrier function [23]. Palmitic acid, a common long-chain saturated fatty acid found in plant and animal fats, is crucial for the skin’s stratum corneum lipids, helping to maintain a healthy skin barrier, preventing infections and moisture loss [24]. Analytical results indicated that both compounds were abundant and stable within ApiRegenin [17]. Therefore, they have been preliminarily selected as non-protein markers for the quality standards of ApiRegenin. To validate their suitability, 12 batches of ApiRegenin, produced at different times, were randomly selected, and the contents of nicotinamide and palmitic acid were measured. As shown in Figure 1A, both compounds were consistently detected across all batches, with relatively stable levels of inter-batch variations, supporting the amounts of nicotinamide and palmitic acid as non-protein biomarkers in the quality control of ApiRegenin.

For the protein components, IGF and fibronectin were chosen as the biomarkers. IGF is one of the most abundant growth factors in PRP and ApiRegenin. Fibronectin, a large glycoprotein in the extracellular matrix, acts as a critical molecular bridge for cell–matrix interactions. Fibronectin promotes wound healing by supporting endothelial cell adhesion and migration to drive angiogenesis, while also facilitating keratinocyte spreading and directional movement for re-epithelialization [25]. To assess the appropriateness of these biomarkers, 12 batches of ApiRegenin, prepared at different times, were analyzed for IGF and fibronectin contents. As illustrated in Figure 1A, both proteins were reliably detected in all batches, with consistent concentrations and no statistically significant variation in batch-to-batch differences. These findings suggest that IGF and fibronectin may serve as potential candidate protein biomarkers for the standardization of ApiRegenin. Based on the determined content, the proposed release criteria limit for 1 g dried powder of ApiRegenin is no less than 0.16 ± 0.03 mg for nicotinamide, 14.60 ± 1.80 mg for palmitic acid, 2.95 ± 0.43 µg for IGF, and 1.12 ± 0.24 mg for fibronectin for assessing the quality of ApiRegenin.

Bioassays are a crucial analytical tool for measuring a product’s biological response in living systems, directly revealing efficacy beyond mere physicochemical attributes. The true value of ApiRegenin can be assessed through the functional responses of cultured cells, such as fibroblasts, providing precise numerical values for the “biological potency” of each batch. Additionally, bioassays serve as critical stability indicators, helping determine the product’s shelf life and support stable supply [26].

The biological activity of ApiRegenin was evaluated through a combination of functional assays. In the DNA-transfected cultures, the luciferase reporter assays, driven by HRE (Figure 1B) and NF-κB (Figure 1C) response elements, were used to quantify the effects on these signaling pathways, while MTT assays (Figure 1D) were conducted in parallel to rule out cytotoxicity as a confounding factor. The dose–response curves were generated using different concentrations of ApiRegenin. The values of EC_50_ (half-maximal effective concentration) were established in parallel with their corresponding controls, i.e., tBHQ in pHRE-Luc assay, dexamethasone in pNF-kB-Luc assay, and VEGF in cell growth assay. The quantitative analysis yielded EC_50_ values for ApiRegenin of 9.05 ± 1.22 mg/mL for pHRE-Luc, 7.85 ± 0.94 mg/mL for pNF-kB-Luc, and 7.33 ± 0.93 mg/mL for cell viability. The high reproducibility of these results underpins the development of a reliable potency assay for quality control and regulatory approval.

### 3.2. ApiRegenin Accelerates In Vivo Diabetic Wound Healing

**Figure 2:**
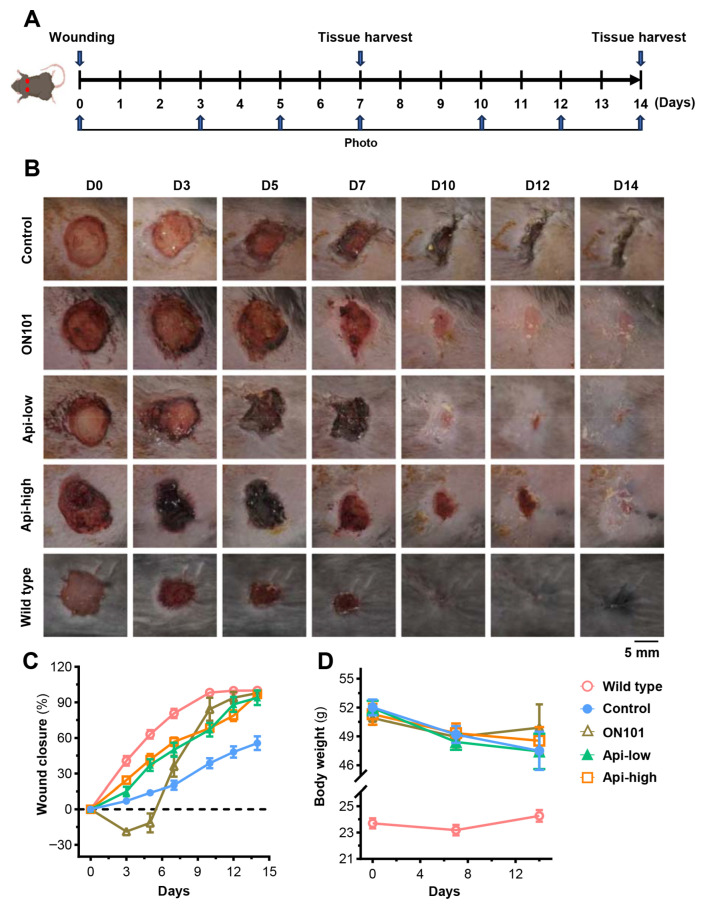
ApiRegenin accelerates in vivo diabetic wound healing. (**A**) Two full-thickness wounds were created on the dorsum. The mice were divided into five treatment groups (*n* = 12): PBS (control); ApiRegenin at 40 mg/mL (Api-low); ApiRegenin at 200 mg/mL (Api-high); ON101 at 1250 mg/mL (ON101); and wild-type mice (wild type). Treatments were administered daily. (**B**) Representative images of the wounds in mice receiving different treatments from days 0 to 14 are shown. (**C**) Relative wound closures are shown at different times using ImageJ software. (**D**) Body weights are shown at different times. Data are expressed as mean ± SEM based on animal-level replicates (*n* = 6).

The therapeutic impact of ApiRegenin on skin wounds was evaluated using a full-thickness wound (8 mm diameter) model of *db/db* and wild-type mice. The tissue collection was performed on day 7 and 14 (Figure 2A). The injury sites were carefully observed for two weeks after the treatment, with photographs taken every 2–3 days. The results showed that wound closure was observed by day 3 in the high-dose ApiRegenin (Api-high), low-dose ApiRegenin (Api-low), control, and wild-type groups. The most prominent increases were seen in the Api-high and wild-type groups, reaching 24.5% and 40.8%, respectively. Conversely, the positive control (ON101) showed a 18.7% reduction in wound closure on day 3, indicating a larger wound area than on day 0; this effect may be associated with aggravated inflammation. Compared to day 5, the healing rates of all groups were significantly improved by day 7, with the healing rates of the Api-high and Api-low groups gradually approaching each other but remaining markedly elevated compared with the control group. This result might be attributed to the role of ApiRegenin in promoting angiogenesis. By day 12, the healing rate of the wild-type group approached 100%, while the healing rates of the Api-low and ON101 groups were both above 88%, indicating that ApiRegenin and ON101 promoted epidermal regeneration. By day 14, wounds in the Api-high, Api-low, ON101, and wild-type groups had almost completely healed. However, the healing rate of the control group was less than 60% (Figure 2B,C). Thus, ApiRegenin has a significant advantage in promoting the healing of chronic diabetic wounds.

The body weight and blood glucose levels of the mice were recorded and analyzed. By day 7, all groups of mice exhibited varying degrees of weight loss compared to day 0, indicating that the presence of a wound had an impact on their growth. As the wounds gradually healed, the trend of weight loss was alleviated by day 14, with the wild-type and ON101 groups showing a trend of recovery, followed by the Api-high group, which returned to the level observed on day 7 (Figure 2D). Random blood glucose measurements showed that blood glucose levels in all groups except the wild-type group stayed above 16.7 mmol/L (Figure S1), confirming the successful establishment of the diabetic mouse model.

### 3.3. ApiRegenin Accelerates Histological Regeneration and Remodeling of Diabetic Wounds

**Figure 3:**
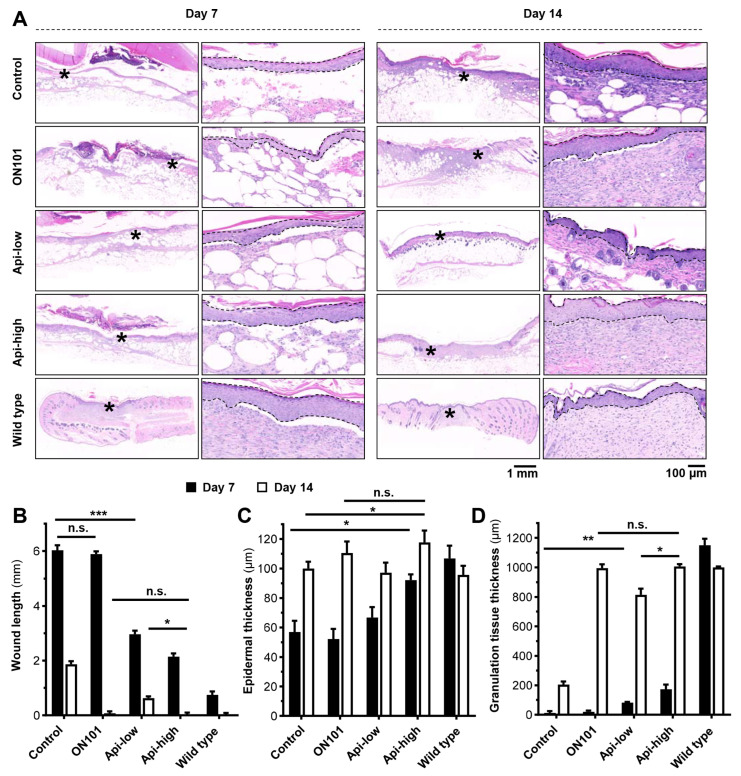
Hematoxylin and eosin staining analysis of diabetic wounds. (**A**) On days 7 and 14, wound skin was harvested, fixed, dehydrated, cleared, and embedded for sectioning. The sections were then subjected to H&E staining, mounted, and observed under a light microscope. Representative images of H&E wound tissues following various treatments are shown. Scale bars = 1 mm (low magnification) and 100 µm (high magnification). The black dashed lines outline the dermo-epidermal junction separating the neo-epidermis from the underlying dermis. Cell nuclei are stained blue-purple by hematoxylin, and the cytoplasm/extracellular matrix is stained pink-red by eosin. (**B**) Wound length was determined as the longest linear distance across the wound surface. (**C**) Epidermal thickness was measured as the vertical distance between the two dashed lines and (**D**) granulation tissue thickness was measured as the average vertical thickness of the granulation tissue layer in each group on days 7 and 14 using Image-Pro Plus software. Data are expressed as mean ± SEM based on animal-level replicates (*n* = 4). * *p* < 0.05; ** *p* < 0.01; *** *p* < 0.001; n.s.: no significant difference.

H&E staining was conducted to evaluate the therapeutic effects of ApiRegenin on the diabetic wounds from a histological perspective (Figure 3A). The treatments of ApiRegenin and ON101 at 14 days resulted in robust skin regeneration. Consistent with the wound closure results, the wound lengths of the control group exhibited retarded reduction (Figure 3B). On the contrary, the wound length of the Api-high group decreased rapidly from 8 mm on day 0 to 2.1 mm on day 7, which was as low as 26.3% of the initial wound size, and wound lengths of 75.4%, 73.6%, 37.1%, and 0.94% were identified from that of control, ON101, Api-low, and wild-type groups, respectively. Re-epithelialization is essential for the restoration of the skin barrier to minimize water loss and infection risk. Api-high treatment resulted in the swiftest epidermis regeneration among all groups, followed by the wild-type and ON101 groups. More specifically, the epidermal thickness of the Api-high group was measured to be 92.25 µm on day 7, higher than the other groups, except the wild-type group. On day 14, the renascent epidermal thickness in the Api-high group further increased to 117.75 µm, which was significantly superior to the control group (*p* < 0.05) but resembled that of the wild-type group (*p* > 0.05), indicating a robust epidermal maturation (Figure 3C).

Granulation tissue, which consists of connective tissue and microvessels, constitutes the preliminary nascent tissue formed during the wound-healing cascade. Wound healing can occur via primary intention or secondary intention and defect healing through secondary intention, during which wounds are typically filled with a dense granulation tissue matrix to support tissue repair. The granulation tissue thickness of the Api-high group was as high as 173.33 µm on day 7, and the wounds were covered with intact and seamless epithelium on day 14, as were those of wild-type mice (Figure 3D). In sharp contrast, the thicknesses of the granulation tissues in the control and ON101 groups were 12.67 µm and 20.67 µm on day 7, respectively. In addition, the wounds of the ON101 and Api-low groups were basically re-epithelialized on day 14, yet there remained improper distinction and adhesion between the epidermis and dermis (Figure 3D).

**Figure 4:**
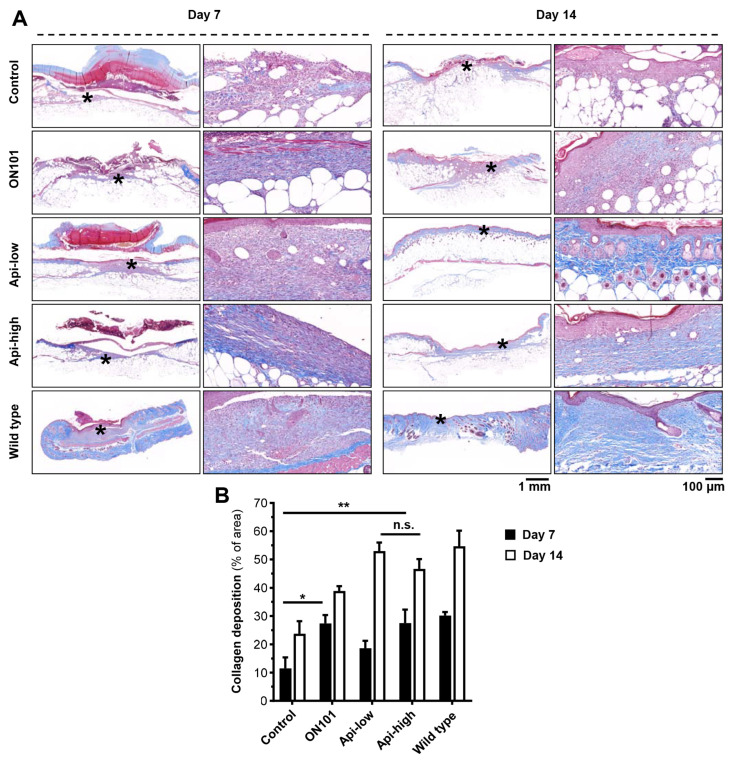
Masson’s trichrome staining analysis of diabetic wounds. (**A**) On days 7 and 14, wound skin was harvested, fixed, dehydrated, cleared, and embedded for sectioning. The sections were then subjected to Masson staining, mounted, and observed under a light microscope. Representative images of wound tissues following various treatments are shown. Scale bars = 1 mm (low magnification) and 100 µm (high magnification). Collagen fibers are stained blue, cell nuclei are stained black, and the cytoplasm/keratin is stained red. (**B**) The percentage of collagen fibers was calculated as the ratio of positively stained area to the total visual field area using Image-Pro Plus software. Data are expressed as mean ± SEM based on animal-level replicates (*n* = 4). * *p* < 0.05; ** *p* < 0.01; n.s.: no significant difference.

Masson’s trichrome staining was employed to visualize the synthesis and accumulation of collagen in the wounds, serving as the basis of the extracellular matrix for tissue regeneration. The collagen fibers in the Api-high group were denser and more orderly in arrangement on day 14 in comparison with the other groups, suggesting the conversion of the granulation tissue into collagen-abundant hypocellular dermal tissue (Figure 4A). In addition, the wild-type group exhibited the highest collagen deposition at both time points of the wound-healing process, but collagen deposition in the ApiRegenin-treated groups, both Api-low and Api-high, was still significantly higher than that of the control group (Figure 4B). Collectively, ApiRegenin substantially accelerated the regeneration and remodeling of diabetic wounds, as recognized by the staining of H&E and Masson.

### 3.4. Neovascularization and Macrophage Polarization In Vivo

**Figure 5:**
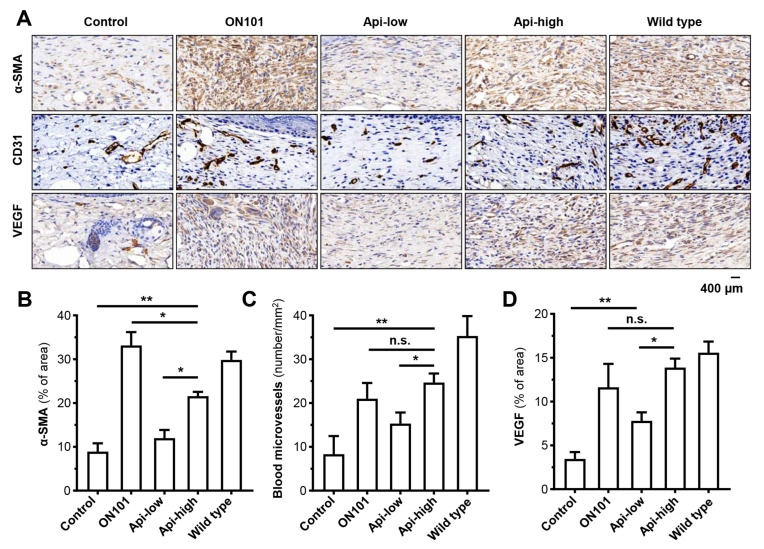
Immunohistochemical detection of α-SMA, CD31 and VEGF on diabetic wounds. (**A**) On day 14, wound skin was harvested, fixed, dehydrated, cleared, and embedded for sectioning. The sections were then subjected to staining and observed under the microscope. Representative images of α-SMA, CD31 and VEGF by IHC on wound tissues following various treatments are shown. Scale bar = 400 µm. Positive expression of the target proteins is stained brown (DAB), and cell nuclei are counterstained blue (hematoxylin). (**B**) Corresponding quantification of α-SMA expression per microscopic field. (**C**) Corresponding quantification of CD31 expression per microscopic field corresponding to the number of microvessels. (**D**) Corresponding quantification of VEGF expression per microscopic field using Image-Pro Plus software. Data are expressed as mean ± SEM based on animal-level replicates (*n* = 4). * *p* < 0.05; ** *p* < 0.01 n.s.: no significant difference.

Tissue regeneration relies heavily on the establishment of a functional neovascular network, which is critical during the proliferation and remodeling phases as it delivers essential oxygen, nutrients, and cells to the repair sites. Blood vessel structure is primarily composed of vascular endothelial cells and smooth muscle cells, which can be specifically labeled by using CD31 and α-SMA antibodies, respectively, as well as VEGF, a key pro-angiogenic factor, to assess the status and maturity of neovascularization. To systematically evaluate angiogenesis on day 14, corresponding quantification of immunofluorescence staining was performed (Figure 5A). The Api-high group exhibited the most prominent α-SMA-positive signals, with significantly greater intensity and distribution compared to the ON101 group and Api-low group on day 14 (Figure 5B). These findings suggest that the API-high group contained more structurally intact and mature vessels with fully formed walls, rather than only early endothelial cell cords. The CD31 staining results further quantified the density of newly formed blood vessels (Figure 5C). All groups showed a higher number of new vessels than the control. Among them, the API-high and ON101 groups demonstrated a statistically significant increase, as compared to control. In addition, the wild-type group showed the most pronounced pro-angiogenic effect and the highest neovessel density. At the molecular level, the expression of VEGF, a key pro-angiogenic factor, was examined. As presented in Figure 5D, the level of VEGF in skin tissue followed a trend with the vascular density, as indicated by CD31, and the API-high group displayed the highest VEGF expression, excluding the wild-type group. From a signaling perspective, this suggested that ApiRegenin might promote cell proliferation by upregulating VEGF expression and subsequently activating downstream pathways. These results indicated that ApiRegenin, particularly at a high dose, effectively stimulated the formation and maturation of new blood vessels by upregulating VEGF expression and promoting the proliferation of cells. This process provides crucial blood supply support for tissue regeneration, thereby accelerating the overall repair process.

### 3.5. RNA Sequencing (RNA-Seq) and Bioinformatic Analysis

**Figure 6:**
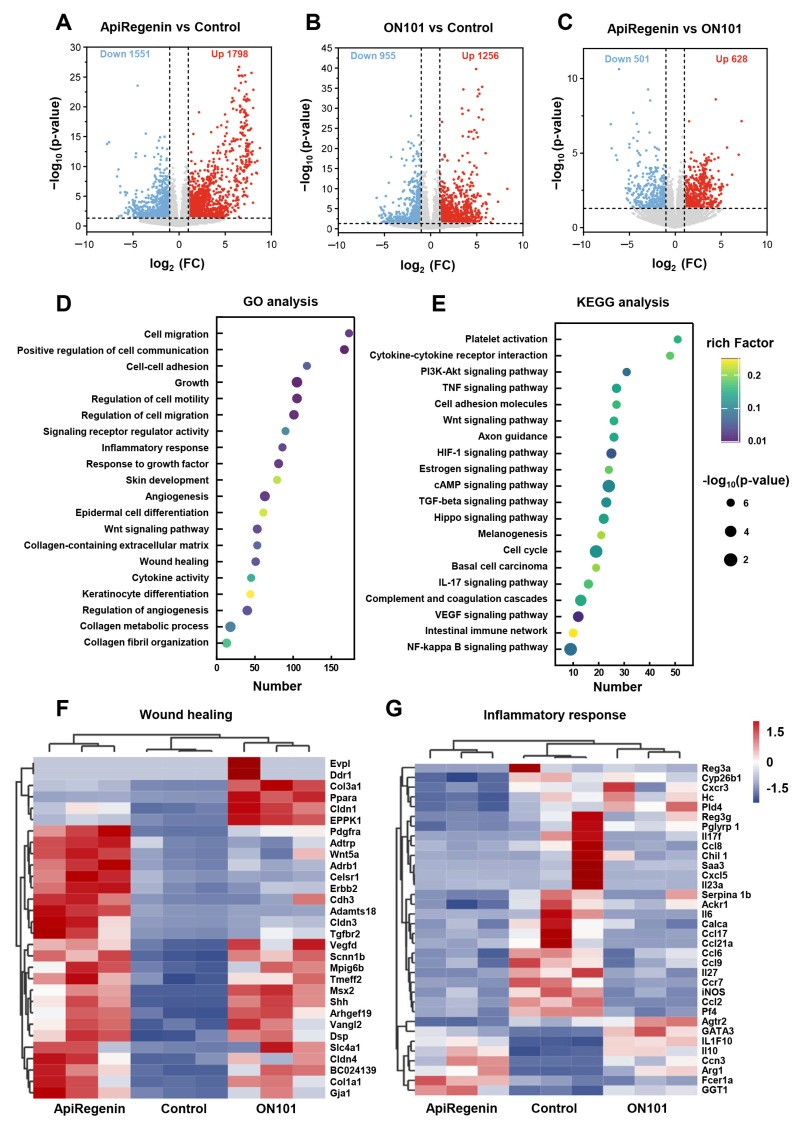
RNA sequencing and bioinformatic analysis on diabetic wounds. Volcano plot analysis of the identified upregulated and downregulated genes between (**A**) ApiRegenin vs. control, (**B**) ON101 vs. control and (**C**) ApiRegenin vs. ON101. The numbers of up- or downregulated targets are shown. (**D**) The GO enrichment of ApiRegenin-treated mice genes is shown. (**E**) The KEGG enrichment pathway of ApiRegenin-treated mice is shown. (**F**) Heatmaps of wound-healing-related genes in control, ON101 and ApiRegenin groups. (**G**) Heatmaps of inflammation-related genes in control, ON101 and ApiRegenin groups.

To further understand the regeneration-promoting mechanisms of ApiRegenin, the tissues on day 14 from the Api-high, ON101, and control groups were collected for transcriptomics analysis. ApiRegenin-treated mice exhibited significantly different gene expression patterns from those of PBS-treated mice in unguided PCA (Figure S2A). The identified genes from three independent replicates exhibited substantial overlaps, demonstrating favorable stability and reliability throughout sample detection and data processing procedures. The Venn diagram illustrates the counts of differentially quantified genes and commonly expressed genes across the three experimental groups (Figure S2B). A total of 21,220, 20,460 and 19,999 genes were identified in the treatments of ApiRegenin, ON101 and control, respectively. Among all these proteins, over 90% (18,762) of the genes were shared by the three groups, demonstrating that a large set of overlapping proteins was detected, which validated the robustness of gene maps to some extent. Additionally, the resulting volcano plot of these overlapping genes between these groups is given. A total of 3349 significant differential genes were obtained, accounting for 17.4% of all detected genes on ApiRegenin vs. control. Among these genes, 1798 and 1551 showed significant up- and downregulation, respectively (Figure 6A). Notably, the differential gene expression profiling between the ON101 group and the control group generated highly consistent findings, which is a promising observation (Figure 6B). Comparison of the differentially expressed genes between ON101 and ApiRegenin revealed that the proportion of divergent genes was less than 5% (Figure 6C). This strongly suggested that ON101 and ApiRegenin could likely share similar pathways in promoting chronic diabetic wound healing.

GO enrichment analysis suggested that ApiRegenin treatment positively regulated wound-healing-related genes, including angiogenesis, cell migration, collagen organization, inflammatory response and extracellular matrix formation (Figure 6D). KEGG pathway analysis suggested that the PI3K-Akt, HIF-1, and TNF signaling pathways—cascades often associated with macrophage polarization—were highly associated with the exploratory transcriptomic signature of ApiRegenin (Figure 6E). The previous literature indicates that the mechanism by which ON101 promotes wound healing is linked to facilitating the transition of macrophages from the M1 to the M2 phenotype [22]. This finding further supported the similarity between ApiRegenin and ON101 in promoting chronic diabetic wound healing, as both likely function through modulating this key aspect of macrophage polarization. The polarization phenotype of macrophages infiltrating the injured region represents a vital index for evaluating the inflammatory reaction.

The expression of wound-healing-related genes was analyzed, revealing that transcripts, such as *Vegf*, *Col3a1*, *Col1a1*, *Pdgf*, and *Tgfb*, were significantly upregulated in both ApiRegenin and ON101 groups (Figure 6F). M1-phenotype macrophages facilitate inflammatory responses via the secretion of pro-inflammatory cytokines, whereas M2-phenotype macrophages inhibit inflammation by releasing anti-inflammatory cytokines, enhancing nerve regeneration, and further boosting angiogenesis. Moreover, inflammatory-related genes were also enriched here (Figure 6G). The results showed that M1-related transcripts, such as *Il6*, *Inos*, *Ccl2*, and *Il23*, were significantly upregulated in the control group, whereas M2-related transcripts, including *Il10*, *Arg1*, *Gata3*, *Ccn3*, and *Fcer1a,* exhibited markedly elevated expression levels in both the ApiRegenin and ON101 groups.

**Figure 7:**
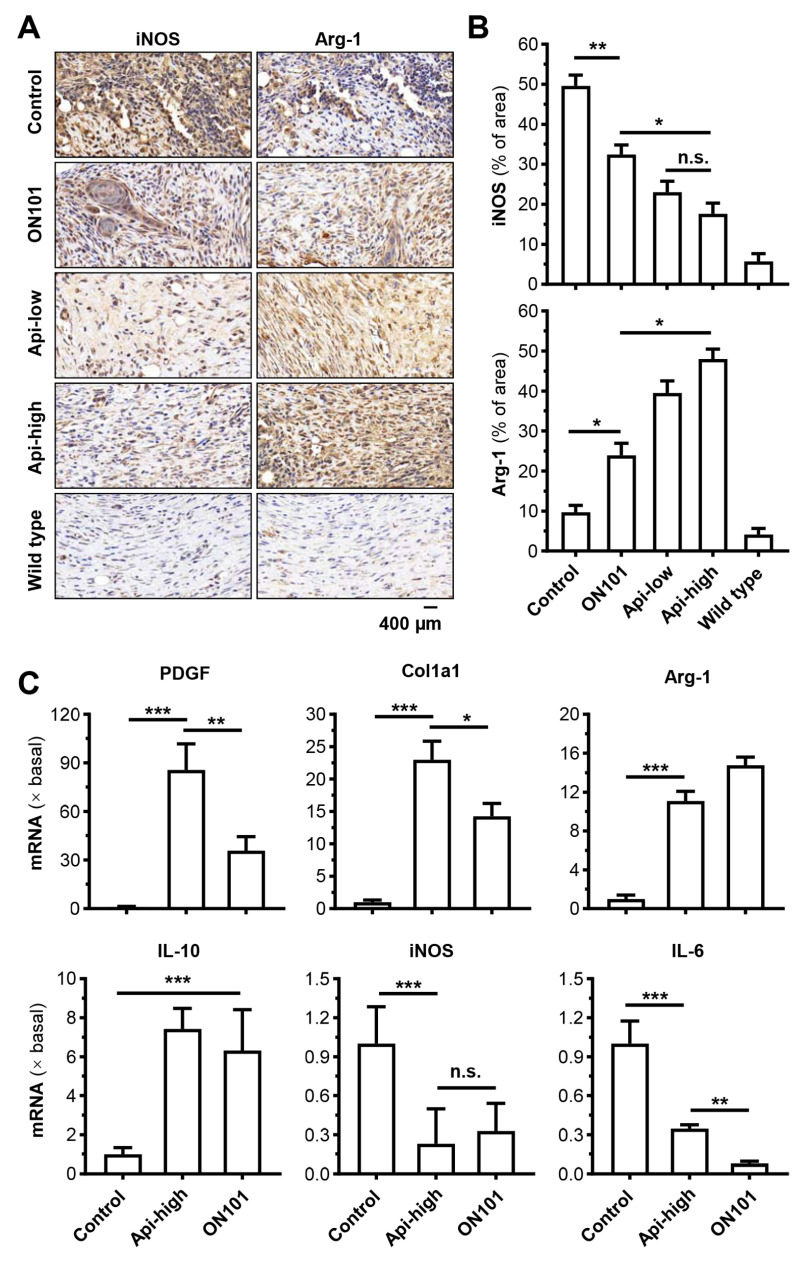
ApiRegenein induces the polarization of macrophages. (**A**) On day 14, wound skin was harvested, fixed, dehydrated, cleared, and embedded for sectioning. The sections were then subjected to staining and observed under the microscope. Representative images of Arg-1 and iNOS by IHC on wound tissues following various treatments are shown. Scale bar = 400 µm. Positive expression of the target proteins is stained brown (DAB), and cell nuclei are counterstained blue (hematoxylin). (**B**) Corresponding quantification of Arg-1 and iNOS expressions per microscopic field using Image-Pro Plus software. (**C**) The mRNA levels of PDGF, Col1a1, Arg-1, IL-10, iNOS, and IL-6 were measured in the skin tissues of the control, ON101 and Api-high groups on day 14. The values are expressed in the fold of change to the normalized basal activity set at 1. Data are expressed as mean ± SEM based on animal-level replicates (*n* = 4). * *p* < 0.05; ** *p* < 0.01; *** *p* < 0.001; n.s.: no significant difference.

Further analysis was performed to validate the RNA-seq findings. Specifically, iNOS and Arg-1 were selected as representative surface markers for M1 and M2 macrophages via immunofluorescence staining in wound tissue sections, respectively. As depicted in Figure 7A,B, the Api-high group exhibited a notably reduced proportion of iNOS-positive expression and a markedly elevated ratio of Arg-1-positive expression within wound-infiltrating cells on day 14 relative to the other groups, suggestive of an attenuated inflammatory microenvironment. Additionally, the percentage of M2 macrophages was found to exceed that of M1 macrophages in wounds treated with ApiRegenin, whereas M1 macrophages constituted the dominant macrophage phenotype in both the ON101 and control groups. These findings demonstrated that inflammatory activation persisted in untreated wounds, while ApiRegenin was associated with a mitigation of the local inflammatory response. Furthermore, ApiRegenin treatment correlated with a remodeling of the tissue microenvironment from a pro-inflammatory to a pro-reparative state, potentially by influencing macrophage polarization marker shifts toward an M2-like phenotype and supporting the release of anti-inflammatory cytokines.

Using RT-PCR, the expressions of six key genes (*Col1a1*, *Pdgf*, *Il6*, *Inos*, *Il10*, and *Arg1*) were examined across the control, Api-high, and ON101 groups. The results demonstrated a consistent pattern; specifically, the mRNA transcript levels of *Col1a1*, *Pdgf*, *Il10*, and *Arg1* were markedly elevated in both the Api-high and ON101 groups, whereas *Il6* and *Inos* were significantly downregulated (Figure 7C). These transcript-level validations confirmed the reliability of our initial RNA-seq results. Taken together, these preclinical data suggest that ApiRegenin may modulate transcriptional responses associated with the HIF-1 and TNF signaling pathways, correlating with shifts toward M2-like macrophage markers, transcriptomic angiogenic signatures, and cell migration in wound tissue, thus ultimately contributing to the accelerated healing progression of diabetic wounds in this experimental model.

## 4. Discussion

The present study explores the preclinical evaluation of ApriRegenin, an optimized bioactive formulation derived from deer PRP, as a potential experimental candidate for accelerating healing, particularly in diabetic mouse wounds. Several lines of evidence support this notion. First, the quality control biomarkers, i.e., nicotinamide, palmitic acid, IGF, and fibrinogen have been established; these biomarkers are consistent across multiple batches of production. Second, functional standards have been established in cultured cells. By employing reporter gene constructs targeting HRE and NF-κB pathways, the potential capacity of ApiRegenin to modulate downstream transcript networks related to angiogenesis and inflammation has been demonstrated. The EC_50_ values could serve as quantifiable indicators of biological efficacy, establishing a direct link between product attributes and relevant biological effects. By ensuring batch-to-batch compositional consistency, this approach provides a foundation for reproducible therapeutic efficacy. Third, the efficacy of ApiRegenin has been illustrated in diabetic wounds in mice. The experimental wound achieved advanced closure after 12 days of ApiRegenin application, and the tissue repair process was supported by histological thickness and its corresponding gene and protein expression profiles. Lastly, potential mechanistic candidate cascades in the wound skin were proposed based on exploratory transcriptomic analysis.

Diabetic wound healing is characterized by a disruption of the normal, highly coordinated repair process. Persistent hyperglycemia leads to impaired skin barrier function, chronic inflammation, oxidative stress, and microvascular damage, ultimately resulting in delayed or non-healing wounds [27]. A hallmark of diabetic wounds is the persistent presence of pro-inflammatory M1 macrophages that perpetuate inflammation and tissue destruction [28]. Here, ApiRegenin was observed to modulate this dysregulated immune response in a preclinical setting. Indeed, the presented KEGG pathway analysis uncovered notable enrichment of the TNF signaling pathway and its corresponding downstream regulatory molecules. In the skin of ApiRegenin-treated mice, M1 markers (IL-6, iNOS) were significantly downregulated, while M2 markers (IL-10, Arg-1) were notably upregulated, indicating a phenotypic marker shift from pro-inflammatory to pro-reparative macrophage signatures. This transition is critical for wound healing because M2 macrophages promote resolution of inflammation, angiogenesis, and fibroblast proliferation. Notably, this immunomodulatory effect of ApiRegenin was in line with that of ON101, a known macrophage modulator, further validating the relevance of this pathway profile [26]. By influencing local immune dysregulation, ApiRegenin creates a favorable microenvironment that facilitates the transition from chronic inflammation to regenerative repair.

Beyond immune modulation, ApiRegenin was associated with the enrichment of key regenerative signaling networks. In a hypoxic environment, a characteristic of diabetic wounds, a significant enrichment of the HIF-1 signaling pathway was suggested by bioinformatics data. Thus, ApiRegenin in the wound site was likely to stabilize HIF-1α, thereby correlating with the transcription of subsequent wound-healing-related genes, most notably VEGF. Both RNA sequencing and RT-PCR confirmed the concurrent upregulation of VEGF and PDGF transcripts, establishing a direct link between pathway activation and functional outcome. Concurrently, ApiRegenin might also interact with the PI3K-Akt signaling pathway, which supports cell survival and growth. This activation may enhance the responsiveness of keratinocytes and endothelial cells to growth factors, amplifying proliferative and migratory signals.

Effective tissue repair ultimately requires restoration of skin structure and mechanical integrity. In the ApiRegenin-treated groups, significant upregulation of transcripts encoding collagen (Col1a1, Col3a1) and TGF-β was observed, indicating enhanced extracellular matrix synthesis and remodeling. This finding is particularly relevant, given that diabetic wounds are characterized by insufficient collagen deposition and poor matrix quality [29]. Furthermore, the proteomic analysis of ApiRegenin revealed the enrichment of proteins related to the focal adhesion pathway, including fibronectin, providing a mechanistic basis for improved cell–ECM interactions [17]. The increased fibronectin deposition in the wound sites could facilitate adhesion, spreading, and migration of fibroblasts and keratinocytes at the wound bed. Thus, ApiRegenin not only stimulates ECM production but also promotes its assembly into a functional provisional matrix, accelerating tissue reconstruction while potentially reducing the risk of abnormal scar formation.

Comparative transcriptomic analysis revealed both convergent and divergent mechanisms between ApiRegenin and ON101 in promoting diabetic wound healing. Both treatments upregulated genes involved in angiogenesis (VEGF) [30], collagen deposition (Col1a1) [31], and skin barrier repair (Cldn4), confirming their shared therapeutic efficacy. However, their target profiles were substantially different. ON101 preferentially induced genes associated with keratinocyte differentiation and immediate ECM construction, such as Evpl and Col3a1. In contrast, ApiRegenin specifically upregulated those genes encoding signaling molecules and receptors, including PDGF, Erbb2, Wnt5a, as well as the cell adhesion molecule Cdh3 [32,33]. This distinct profile suggested that ApiRegenin primarily acts by modulating intercellular networks within the wound microenvironment. By enhancing receptor expression and activating specific signaling pathways, ApiRegenin might be more effective at coordinating the proliferation, migration, and differentiation of diverse cell types, including fibroblasts and keratinocytes. This mechanism of action confers potential therapeutic utility for complex chronic wounds, as characterized by disrupted cell signaling and impaired cell migration.

Despite these encouraging preclinical results, several translation boundaries remain. First, the candidate pathways suggested by KEGG enrichment (HIF-1, PI3K-Akt, and TNF) are transcript-level associations rather than definitive proofs of functional protein phosphorylation or activation cascades. Direct independent protein-level assays remain required. Second, this study relies entirely on a murine model where wound closure is driven predominantly by panniculus carnosus-mediated contraction rather than the human-like re-epithelialization. Third, as an animal-derived biological product, extensive systemic safety testing, immunogenicity evaluation, and rigorous standardization must be conducted in clinically relevant, non-contracting large-animal models before any clinical use can be considered.

## 5. Conclusions

Here, we have developed ApiRegenin, a deer-derived PRP formulation with a proposed framework for standardized biomarkers and preliminary bioactivities in vitro. Our findings support the notion that ApiRegenin effectively accelerates diabetic wound healing in a mouse model, correlating with an M1-to-M2 macrophage marker shift and the transcript-level upregulation of pathways associated with angiogenesis and ECM remodeling. Compared to ON101, ApiRegenin offers a unique exploratory transcriptomic signature by specifically modulating genes related to intercellular communication and receptor expression. However, these findings remain preclinical and exploratory. ApiRegenin represents a promising experimental candidate that requires further biological standardization, rigorous protein-level mechanistic validation, extensive long-term safety and immunogenicity testing, and evaluation in clinically relevant wound models before its true translational potential can be established.

## Data Availability

The data presented in this study are available upon request from the corresponding author.

## Supplementary Materials

The following supporting information can be downloaded at https://www.mdpi.com/article/10.3390/pharmaceutics18070856/s1. Figure S1: Blood glucose levels of mice. Blood glucose levels at different time points were measured using test strips. Data are expressed as mean ± SEM based on animal-level replicates (*n* = 6).; Figure S2: PCA scores and Venn diagram analysis of identified genes. (A) PCA scores for identified genes in wound tissue from mice of different groups on day 14. Each data point in the plot represents an independent biological sample, positioned according to its scores on the first two principal components (PC1 and PC2). Samples from the two groups are distinguished by different colors or shapes. (B) Venn diagram analysis identified genes in wound tissue from mice of different groups on day 14. The three circles represent the sets of unique genes detected in the control, ON101 and ApiRegenin groups, respectively, with the overlapping region indicating genes common to both groups.
