# Supplementary material for: ApiRegenin, an Animal-Derived Platelet-Rich Plasma Extract, Accelerates Wound Healing of Chronic Diabetic Ulcer in Mice
Source: Pharmaceutics. 2026 Jul 14;18(7):856. doi: 10.3390/pharmaceutics18070856 (PMC13415306; doi:10.3390/pharmaceutics18070856)
Supplement: Supplementary file 1 [file pharmaceutics-18-00856-s001.zip › pharmaceutics-4399906-supplementary.pdf]

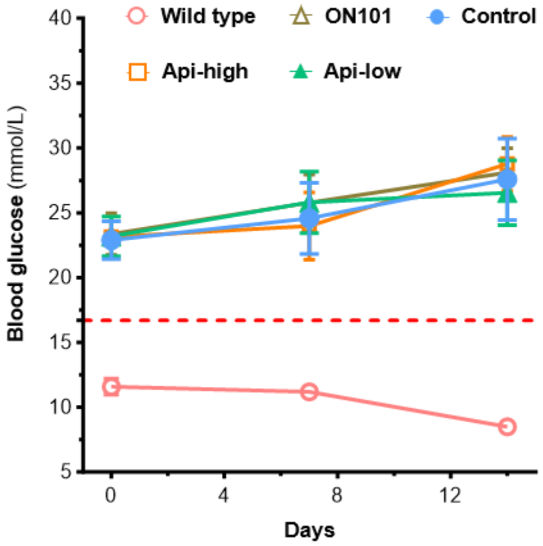

Figure S1. Blood glucose levels of mice.
Blood glucose at different time points were measured using test strips. Data were expressed as mean ± SEM based on animal-level replicates (n = 6).

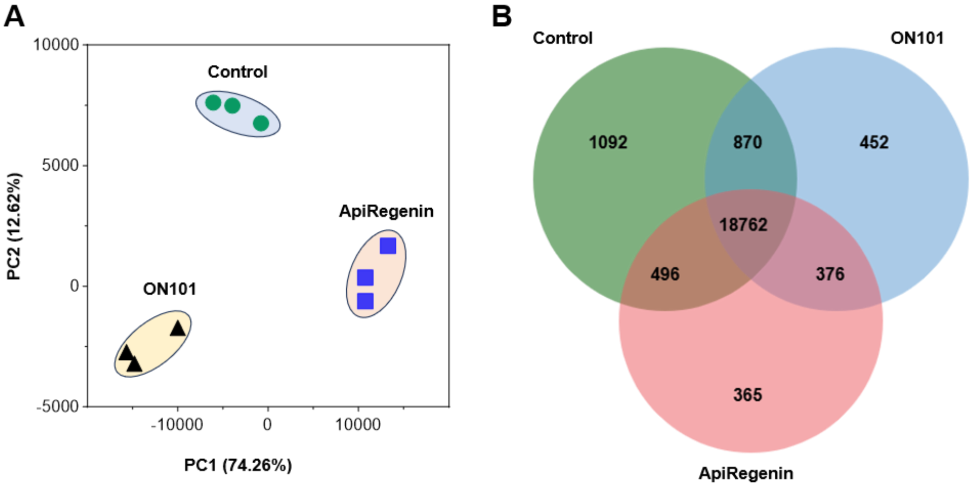

Figure S2. PCA scores and Venn diagram analysis of identified genes. **(A)** PCA scores for identified genes in wound tissue from mice of different groups on day 14. Each data point in the plot repre-sents an independent biological sample, positioned according to its scores on the first two principal components (PC1 and PC2). Samples from the two groups are distinguished by different colors or shapes. **(B)** Venn diagram analysis identified genes in wound tissue from mice of different groups on day 14. The three circles represent the sets of unique genes detected in control, ON101 and ApiRegenin, respectively, with the overlapping region indicating genes common to both groups.
